# A novel recombinant human plasminogen activator: Efficient expression and hereditary stability in transgenic goats and in vitro thrombolytic bioactivity in the milk of transgenic goats

**DOI:** 10.1371/journal.pone.0201788

**Published:** 2018-08-17

**Authors:** Zhengyi He, Rui Lu, Ting Zhang, Lei Jiang, Minya Zhou, Daijin Wu, Yong Cheng

**Affiliations:** College of Veterinary Medicine/Jiangsu Co-innovation Center for Prevention and Control of Important Animal Infectious Diseases and Zoonosis, Yangzhou University, Yangzhou, Jiangsu, China; University of Illinois, UNITED STATES

## Abstract

**Background:**

Thromboses is a rapidly growing medical problem worldwide. Low-cost, high-scale production of thrombotic drugs is needed to meet the demand. The production of biomolecules in transgenic animals might help address this issue. To our knowledge, the expression of recombinant human plasminogen activator (rhPA) in goat mammary glands has never been reported before.

**Methods:**

We constructed a mammary gland–specific expression vector, BLC14/rhPA, which encodes only the essential K2 fibrin-binding and P domains of wild-type tPA (deletion mutant of tPA lacking the F, E, and K1 domains), along with the goat β-lactoglobulin gene signal peptide-coding sequence. The mammary gland–specific expression vector BLC14/rhPA was transfected into goat fetal fibroblast cells by electroporation. After selection for 3 weeks by G418, stably transfected cell colonies were obtained. PCR analysis results indicated that 24 of the resistant clones were transgenic cell lines; of these, 8 lines were selected as the donor cells. The positive cells were starved for 72 h with DMEM/F12 medium containing 0.5% FBS and were then used as do. Finally, 256 reconstructed oocytes were transferred into 26 recipients, and 7 of them became pregnant (pregnancy rate, 26.9%). Two kids were obtained (BP21 and BP22). PCR analysis confirmed that both were transgenic goats. To analyze the heredity of the *rhPA* expressed in BP21 F0 and F1 transgenic goats, the F0 transgenic goat BP21 was mated with a normal male goat to generate an F1 transgenic goat. Enucleated metaphase II (MII) oocytes and positive donor cells were used to reconstruct embryos, which were transplanted into the oviducts of the recipients.

**Results:**

Western blot results showed a specific 39 kDa band. The rhPA expression level in transgenic goat whey was about 78.32 μg/mL by ELISA. Results of ELISA and the *in vitro* thrombolysis test (FAPA) showed that specific activity of the rhPA in the milk of F0 and F1 transgenic goats was 13.3 times higher than that of the reteplase reference material.

**Conclusion:**

Thus, we demonstrated that BLC14/rhPA was reasonably effective for expression in the mammary glands of transgenic goats, and was stably inherited by the offspring. This study provides the basis for the large-scale production of biological pharmaceuticals in transgenic animals. The expression of biopharmaceuticals by transgenic animals can be used for pharmacological research and bioactive analysis, and transgenic goats were demonstrated to be promising animals for the large-scale production of thrombolytic biopharmaceuticals.

## Introduction

Thrombotic diseases are among the most common diseases in humans[1–2]. Cardiovascular disease, especially thromboembolism, is a grave risk to human health, given its steady increase every year. The rates of thrombosis, which includes acute myocardial infarction, brain death, pulmonary embolism, deep vein thrombosis, and peripheral vascular embolization, are very high, and the associated rates of morbidity and mortality are also formidable [1]. According to the World Health Organization, 1 person dies of this disease every 6 s in the world. Its risk is much higher than that of cancer among the top causes of death in humans.

At present, thrombolytic agents have been proved to be the main and most effective treatment approach of such diseases[3–5]. Thrombolytic drugs, including urokinase, streptokinase, and human tissue-type plasminogen activator (tPA), are used in clinical treatment. tPA and its derivatives are considered the most effective thrombolytic agents, and they are mainly used for myocardial infraction, cerebral thrombosis, and other venous thromboembolism diseases[3–6].

Therefore, the development of more efficient, more specific, cheaper, and safer thrombolytic drugs is warranted. Plasminogen activators have been developed till the third generation, and recombinant human plasminogen activator (rhPA) represents the latest among them. It is a key fibrinolysis kinase that plays an important role in dissolving fibrin clots to promote thrombolysis [3–6]. Compared with natural tPA, the deletion mutant lacking the F, E, and K1 domains has the advantages of strong thrombolytic activity (such as highly specific affinity to fibrin), long half-life in the blood, few side-effects (such as systemic bleeding), and low total dose. Therefore, as a representative of the third-generation thrombolytic agents, rhPA is increasingly being used in the clinical setting [6–10].

Recombinant proteins are rapidly gaining popularity as biopharmaceuticals [11–13]. Therefore, considerable attention is being paid to the reduction in the primary cost of recombinant proteins and improvement of their quality by selecting new and more efficient expression systems[11–13]. There are many disadvantages of producing recombinant proteins in bacterial hosts, mammalian cells, yeasts, viruses, transgenic plants, etc., because of poor biological activity, high cost, and poor post-translation modification of proteins [13]. Owing to the high expression level, low cost, ease of purification[6], ideal post-translation modification similar to that of human proteins, and higher biological activity, the mammary gland bioreactor overcomes nearly all the shortcomings and disadvantages of other expression systems. Therefore, medicinal recombinant proteins expressed in transgenic animal mammary gland bioreactors are gaining the attention of researchers and have wide development prospects[14–17]^.^ Animal mammary glands have become the most ideal expression systems to produce recombinant proteins. Many recombinant proteins such as antithrombin (ATryn), human alpha-1 antitrypsin (hAAT), human protein C (hPC), human fibrinogen (hFIB), human serum albumin (hSA), human clotting factors VIII and IX (hF-VIII, IX), tPA, hepatic leukemia factor (hLF), homoglutamic acid (hGlu), superoxide dismutase (SOD), and hormones have been generated in the milk of transgenic animals[14].

Compared to other animals, such as cattle, transgenic goats have many advantages: lower feeding cost, higher milk yield, higher survival rate, longer life, and higher reproductive rate. Using transgenic goats to produce rhPA could greatly improve the recombinant protein production capacity and reduce the cost[14–17]. Thus far, rhPA expression in goat mammary glands has not been reported.

Expressing recombinant protein in the mammary glands of transgenic animals is a very complicated process[11–14]. We constructed the mammary gland–specific expression vector BLC14/rhPA, which can express the variant tPA protein, in which the F, E, K1 domains and the glycosylation site Asp 117 are deleted from the cDNA of wild-type human tPA. The BLC14/rhPA vector was transfected into goat fetal fibroblasts, and somatic cell nuclear transfer (SCNT) and *rhPA* transgenic goat embryo transfer were carried out. The original generation *rhPA* transgenic goats were mated with normal goats to breed *rhPA* transgenic offspring. Two transgenic goats with high and stable expression were obtained (BP21 and BP22). *In vitro* ELISA and fibrin agarose plate assay (FAPA) were used to analyze the rhPA expression level and stability in the milk of the F0 and F1 transgenic goats. We demonstrated that the transgenic goats could effectively express rhPA in their milk. We found that the rhPA expression level was stable throughout the lactation.

## Materials and methods

### Animals

Yangtze River Delta white goats were bought from and maintained at the Research Farm of Yangzhou University, China. Wild-type control goats were produced by normal sexual reproduction. A total of 100 female goats (age: 2 years; average body weight: 45±6.5 kg) were used in this study. All experiments of this study followed the Guiding Principles for the Care and Use of Laboratory Animals, and were approved by the Institutional Animal Care and Use Committee of Yangzhou University, Ministry of Science and Technology of the People’s Republic of China (SYXK2016-0019) [18].

All goats in this study were raised at room temperature (25±2°C, 40%–60% humidity) with 12-hours dark-light cycle. Each goat was given access to food and water freely.

Food was not gave to the goats about 24 hours before surgery to reduce the risk of intestinal tympanites. All the animals were anesthesia by 0.04 mg/kg of atropine subcutaneously, followed by 10 mg/kg of ketamine and 0.3 mg/kg of xylazine intramuscularly before surgery. The goats surgeries were performed in supine position. After the completion of surgery, we give the animal food and water when they began to eat by itself. Cefazolin (25 mg/kg) was administered once a day for seven days after surgery. Goats care was performed by trained animal care staff. Eating habits, animal behaviors, health status were monitored daily for the first 2 weeks.

The goats post-surgery pain were relieved by NSAIDS (non-steroidal anti-inflammatory drugs, such as ibuprofen) and opioids (such as meperidine and morphine). Vegetables was provided once a day within the first week after surgery.

### Construction of mammary gland–specific rhPA gene expression vector

**Fig 1:**
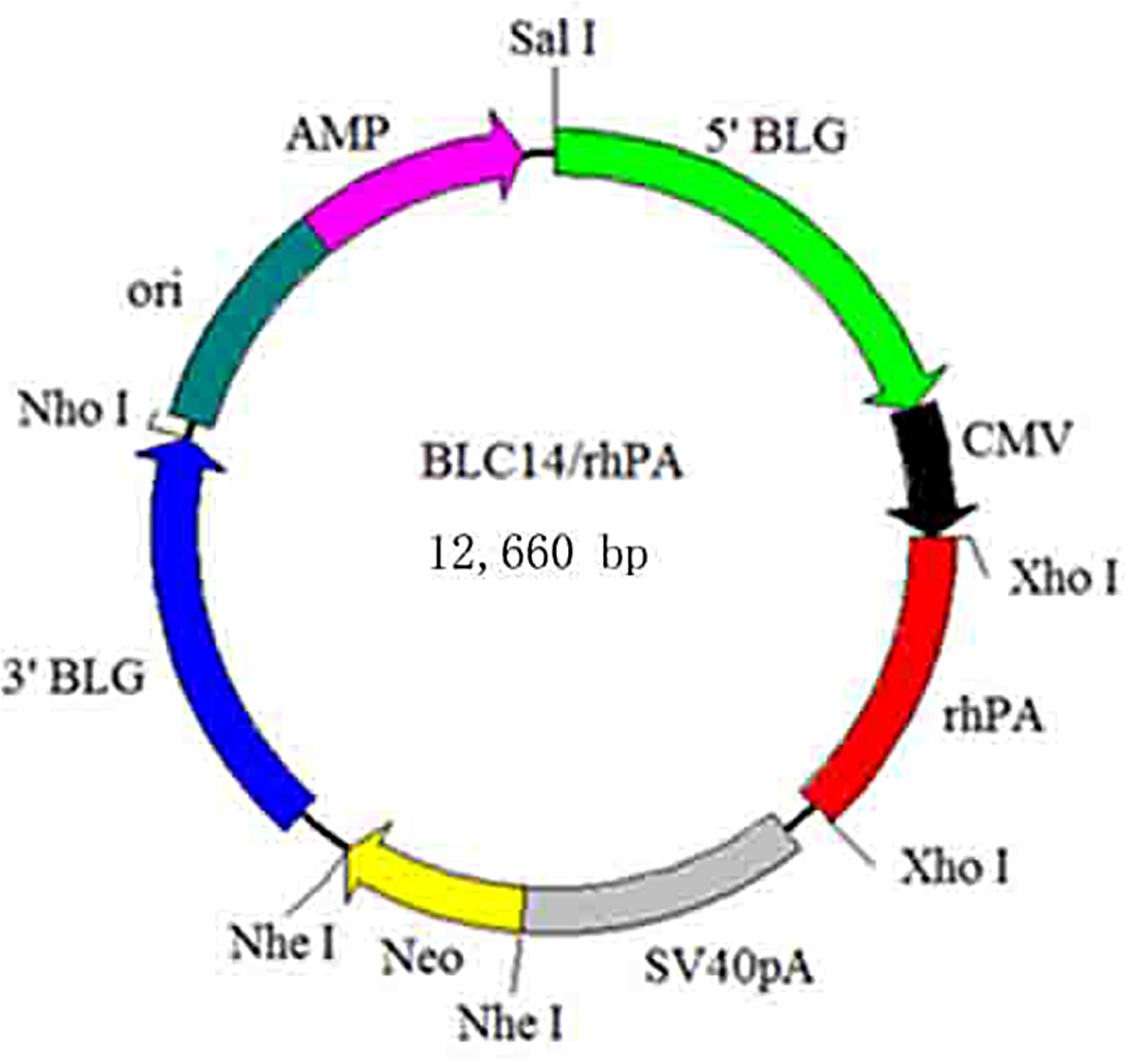
Diagram of the BLC14/rhPA vector. rhPA was cloned into the XhoI site of the BLC14/rhPA vector. BLG promoter: goat β-lactoglobulin; CMV: Human cytomegalovirus immediate-early promoter/enhancer; rhPA: optimized rhPA coding region; Neor: neomycin resistance gene; Loci of primers; SV40: Simian vacuolating virus 40 enhancer, AMP: ampicillin resistance gene.

All the DNA marker were purchased from Takara, Japan. The BLC14 plasmid was previously generated and cloned by the Engineering Research Center for Transgenic Animal Pharmaceutics, Yangzhou University, Jiangsu Province, China. It includes the CMV, rpa, and lactoglobulin promoter regulator, as well as an effective rhPA mammary gland–specific expression vector (deletion mutant of tPA lacking F, E, K1 domains). The vector BLC14/rhPA included the goat β-lactoglobulin gene as the regulatory element, which contained only the essential K2 fibrin-binding domain (to increase the half-life and thrombolytic activity of rhPA) and P-activating plasminogen domain of the wild-type tPA, together with the goat β-lactoglobulin gene signal peptide-coding sequence[2]. All agents were purchased from Sigma Chemicals. The GenBank accession number of wild-type tPA is X77531. The vectors were digested with *Not*I and *Sal*I (Takara, Japan), and the target fragments were purified by the QIAquick Gel Extraction Kit (Qiagen, Germany). Goat fetal fibroblast isolation and culture methods were the same as those described in a previous study. The resulting vector was designated BLC14-CMV-rhPA-Neo (Fig 1).

### Goat fetal fibroblast cell culture and transgene transfection

Approximately 1 × 10^6^ female fetal fibroblast cells were transfected with 5–20 μg/mL of BLC14-CMV-rhPA-Neo purified DNA fragments by electroporation. Then, the cells were transferred to 6-well plates with selective medium containing G418 (600 μg/mL; Amresco Inc., Washington State USA) to screen out rhPA-positive cells. All the normal fetal fibroblasts died 7–10 days after the addition of the screening nutrient solution [18]. The positive transgenic cell lines were detected by PCR. Normal goat fibroblasts were used as the negative control. The positive transgenic cell lines were collected by a cloning cylinder (Sigma-Aldrich, Shanghai China) and transferred to 24-well plates. The transfection cell suspension culture was placed in the 6 wells at 37°C, 5% CO_2_, and saturated humidity conditions. After 48 h, cells were harvested. A quarter of the cells were subjected to PCR analysis, and the rest were cryopreserved in liquid nitrogen.

### Generation of transgenic rhPA goats and analysis of the hereditary stability in F0 and F1 transgenic goats

The *rhPA* transgenic goats were generated through SCNT [18]. The nuclei of the transgenic cells were transferred to enucleated oocytes and electrically fused by a super electro cell fusion generator (EGFE21; Nepa Gene, Chiba-shi Japan). The electrical transfection parameters were as follows: eukaryotes, “⊙”; voltage (V), 250 V; time constant (τ), 280 μs; number of pulses (n), 2.

The reconstructed embryos were activated with 5 μmol/L ionomycin and 7.5 μg/mL cytochalasin B in M16 medium for 5 min, followed by treatment with 2 mmol/L 6-dimethylaminopurine and 7.5 μg/mL cytochalasin B in M16 medium for 5 h. Activated embryos were transferred to synchronous recipient goats, and pregnancy was determined by abdominal ultrasonographic examination 1 month after the SCNT. The average pregnancy in goats is about 150 days. After the birth of the kids, PCR and Southern blot analysis were performed to identify the transgenic ones. The methods of animal oocyte collection, nuclear transfer, embryo transfer, and testing pregnancy status were as described in an earlier study [18]. Ten cell strains of each sex were used as donor cells for SCNT. Several cell lines containing the desired mutant genotype were isolated from pregnant recipients and expanded to be used as donor cells for the next round of SCNT.

### Detection of the rhPA gene in the cell/goat genome

Transgenic goats and cells were identified by PCR and sequencing screening analysis of the extracted genomic DNA. The upstream primer is in the CMV region, and the downstream primer R1 is in the *rhPA*-coding region. The amplification product size is 564 bp, include partial of the CMV and rhPA gene. The primers were designed by Primer 5.0 (CMV/rhPA set), and the primer sequences were as follows: cmv+rpa1, 5′-CGTGGATAGCGGTTTGA-3′; cmv+rpa2, 5′-GAACCCTCCTTTGATGC-3′. PCR cycling was as follows: 95°C for 5 min; 33 cycles of 94°C for 45 s, annealing at 57°C for 45 s, and extension at 72°C for 1 min; and 72°C for 10 min. Sequencing analysis was performed by Sangon (Shanghai, China).

The BLC14-CMV-rhPA-Neo plasmids and the genomic DNA samples extracted from the ears of transgenic and wild-type goats were digested overnight with the restriction enzyme *Bam*HI. The digoxigenin-labeled probe was amplified using the corresponding primer pairs. After agarose gel electrophoresis for 12 h, DNA was transferred to a nylon membrane (Roche, Basel Switzerland) for blotting. The nylon membrane was hybridized with probes for 18 h and incubated with the corresponding antibody for 0.5 h according to the instruction manual of the Southern blot kit (Boster, Wuhan, China). The size of the positive bands was expected to be about 2,693 bp for BLC14-CMV-rhPA-Neo. The *rhPA* primer pairs were used for PCR amplification and as probes for Southern blot analysis.

### Analysis of the hereditary stability of the transgene in the F0 and F1 transgenic goats

To analyze the hereditary stability of the *rhPA* in BP21 F0 and F1 transgenic goats, the F0 transgenic goat BP21 was mated with a normal male goat to generate the F1 transgenic goat.

### Pretreatment of transgenic goat milk

To characterize the expression of rhPA, milk was collected during lactation. Milk of F0 and F1 transgenic goats was pretreated before ELISA, FAPA, PAGE-SDS, and western blot analyses. The goat milk was centrifuged at 12,000 ×*g*/min and 4°C for 30 min. The milk was defatted, and precipitates were removed. The whey was collected; its pH value was adjusted with 50% phosphoric acid and 0.5 mol/L HCl to pH 2.5; and it was centrifuged at 12,000 ×*g*/min and 4°C for 30 min. Next, the supernatant was collected, and the pH value was adjusted with 50% 1 mol/L NaOH to pH 7.5. It was centrifuged at 12,000 ×*g*/min and 4°C for 10 min, and the supernatant was collected again [6]. The final supernatant was filtered by 0.22 μm aperture membrane filtration to remove impurities.

### ELISA for transgenic goat milk rhPA

ELISA was used to determine the rhPA level in transgenic goat milk. ELISA kits (Westtang Biotechnology Co., Ltd., Shanghai China) were used according to the ELISA kit instruction manual. *rhPA* transgenic goat milk whey was immobilized on the ELISA plates as the detecting antigen. The reteplase reference material was used as the positive control, which was purchased from the National Institute for Biological Standards and Control (NIBSC, Potters Bar Britain). Normal non-transgenic goat milk whey was used as the control antigen to exclude the false positive results. PBS (137 mM NaCl, 10 mM Na_2_HPO_4_, 3 mM KCl, 2 mM KH_2_PO_4_, pH 7.4) was used as the blank control. After thorough washing, the complex was mixed with the chromogenic substrate tetramethylbenzidine. The intensity of the color and the rhPA in samples were positively correlated. Absorbance (optical density; OD) was tested by ELISA at 450 nm, and all the concentrations of rhPA in the milk samples were calculated using the regression equations of the standard curves based on their OD values [6].

### In vitro characterization of rhPA thrombolytic activity in transgenic goat milk

The *in vitro* thrombolytic activity of rhPA in F0 and F1 transgenic goat milk was evaluated by FAPA [6]. The *rhPA* transgenic goat milk whey samples were diluted for analysis using PBS. PBS was also used as a solvent with 1.0% agarose gel, 10 mg/mL fibrinogen, and 1 U/mL thrombin. Agarose gel (15 mL) was boiled for melting. When the temperature dropped to 55–60°C, 1 mL fibrinogen was preheated at 37°C, and thrombin was warmed to 42°C. Both were immediately mixed and put into a glass dish. When the solution cooled to room temperature, fibrin-thrombin-agarose solidified to a gel state. Sample wells were drilled in each gel, and each sample well was filled with 20 μL sample solution. Incubation was carried out at a constant temperature of 37°C for 24 h. rhPA activity in the samples was calculated in the regression equations of the standard curves based on the diameter of the transparent zone of the thrombin-dissolving ring [19].

### Evaluation of transgenic goat milk rhPA expression by western blot analyses

We used western blotting to analyze the expression of rhPA in F0 and F1 transgenic goat milk according to previous methods [6]. All the samples were denatured in boiling water (100°C) for 5 min with SDS-PAGE loading buffer. The protein bands were separated under denaturing conditions by 12% SDS-PAGE using Bio-Rad 1.5 mM spacers (denaturing PAGE; Mini-Protean II Electrophoresis Cell; Bio-Rad Laboratories, Hercules, CA, USA) and stained with Coomassie blue. Then, the unstained proteins were electrotransferred to PVDF membranes (0.45 μm; Pall Corp., Beijing China) for western blot analysis. To detect the expression of the rhPA protein, after transfer, the membrane was blocked with 5% (v/v) skimmed milk containing 0.1% Tween-20 for 90 min at 42°C. Then, the membrane was probed overnight at 4°C in the same buffer containing an anti-rhPA primary antibody (1:2000 dilution, against human tPA, mouse monoclonal IgG, sc-59721; Santa Cruz Biotechnology, Santa Cruz, CA, USA). Afterwards, the membrane was incubated with the secondary antibody conjugated with HRP antibody (1:2000 dilution, goat anti-mouse IgG-HRP, sc-2005; Santa Cruz Biotechnology) for 2 h at 37°C. Finally, the membrane was washed thrice with the wash buffer, and immuno detection was carried out with ECL substrate solution (Sangon) according to the manufacturer’s instructions.

## Results

### Construction of the rhPA expression vectors

**Fig 2:**
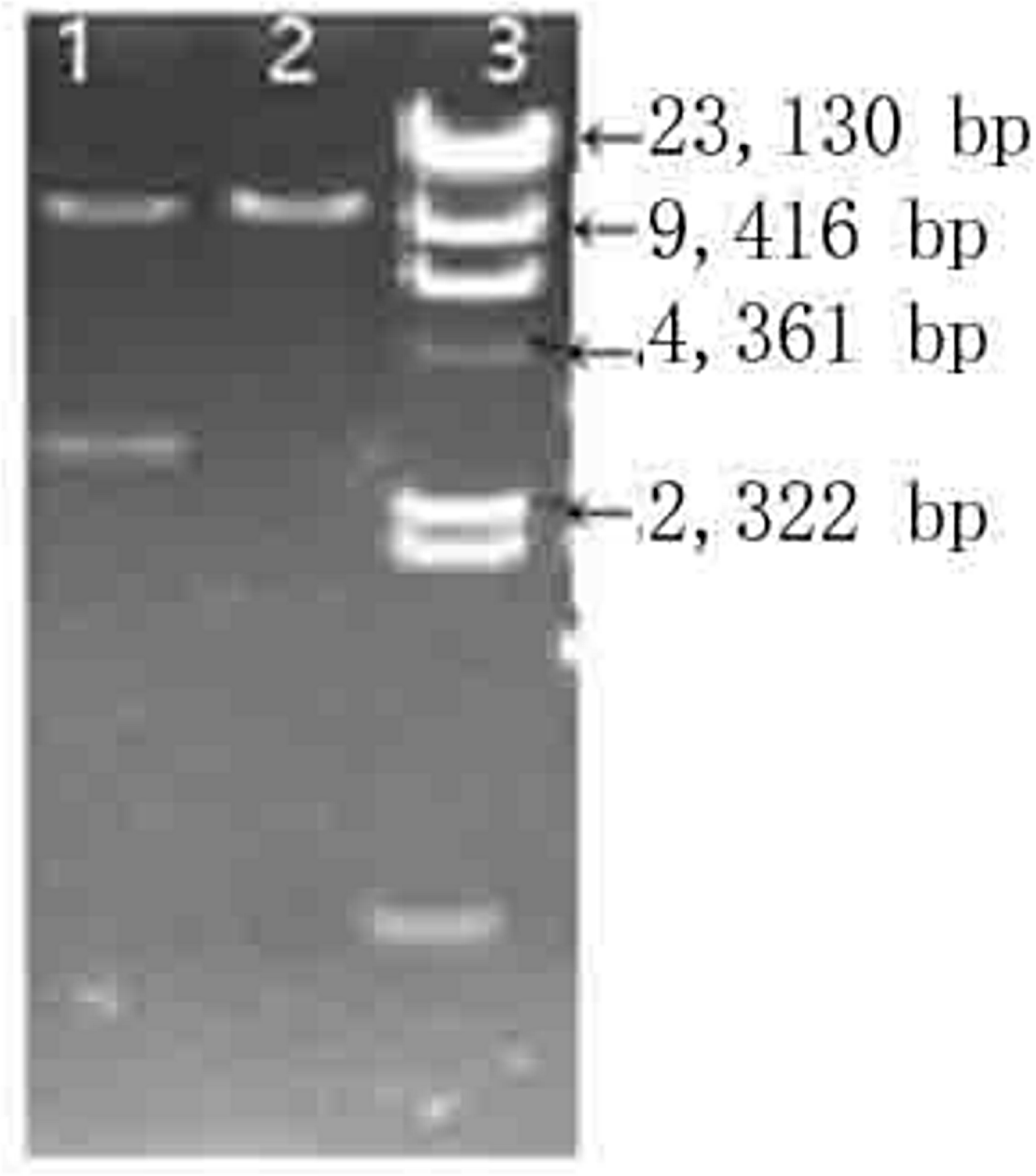
Enzymatic digestion of BLC14/rhPA. 1: BLC14/rhPA plasmid digested by SalI and NotI, 2: BLC14/rhPA fragment for microinjection, 3: λ-Hind III-digested DNA marker.

We successfully constructed the BLC14-CMV-rhPA-Neo vector (Fig 1). The sizes of the recombinant plasmid constructs were confirmed by sequencing. The sequencing result showed that the coding region of the *rhPA* gene was fused in-frame to the upstream region of the vector. We used the *Not*I and *Sal*I double enzyme-digested plasmid BLC14/rhPA. Next, we used a gel recycling and purification kit to obtain a 9.5 kb fragment for microinjection. The results of the agarose gel electrophoresis of the PCR products are shown in (Fig 2).

### Generation of rhPA transgenic goat fetal fibroblasts

**Fig 3:**
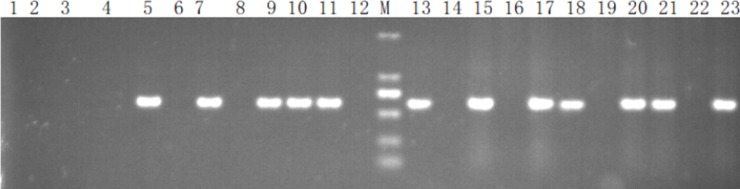
Identification of rhPA in partial transgenic cell lines and goats by PCR. 1 and 2: Double-distilled water as the blank. 3 and 4, PCR amplification products of normal goat cell as the negative control. 5–15: PCR amplification products of rhPA monoclonal cell lines. 17: PCR amplification products of rhPA in the transgenic goat BP21, 18: PCR amplification products of rhPA in the transgenic goat BP22; M: DL2000 DNA marker; 20–21: PCR amplification products of rhPA in the F1 transgenic goats; 22: PCR amplification products of a non-transgenic goat as the negative control; 23: PCR amplification products of the transfected rhPA gene PCR product as the positive control.

**Fig 4:**
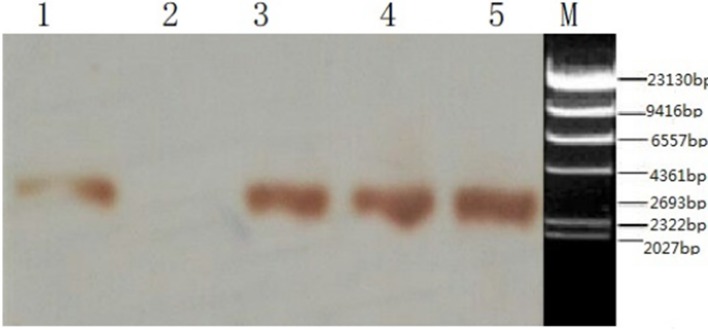
Identification of rhPA in the donor cells and transgenic goats by Southern blot analysis. 1: Southern blot of the rhPA transgenic cell line; 2: Southern blot of a non-transgenic goat as the negative control; 3: Southern blot of rhPA from the transgenic goat BP21; 4: Southern blot of rhPA from the F1 transgenic goats; 5: Southern blot of the BLC14/rhPA plasmid as the positive control,2693bp,M:λ-Hind Ⅲ digest DNA Marker, Takara Bio-joint-stock Company, Japan.

Female goat fetal fibroblast cells (cell line: GFFC-N37) isolated at our laboratory were transfected with the purified BLC14-CMV-rhPA-Neo DNA fragments. Then, 37 isolated colonies analyzed by single-cell amplification were selected with 600 ng/μL G418 for 10 days by PCR (Fig 3). Results showed 8 colonies that had integrated the *rhPA* gene. Colonies of #9 and #30 were chosen for SCNT because they had better quality and viability. The transgenic *rhPA* goat fetal fibroblasts were analyzed by PCR and BLAST. Agarose gel electrophoresis was subsequently conducted, and the 564 bp PCR product was identified (Fig 4).

### Generation of rhPA transgenic goats via SCNT and generation of F1 transgenic goats

To produce *rhPA* transgenic goats, 10 well-grown *rhPA* transgenic cell strains were selected for use as donor cells for SCNT. As shown in (Fig 3), eight cell strains carried the *rhPA* gene. We obtained 438 eggs from 100 female goats, and 256 embryos were reconstructed and transferred into 26 synchronized recipients. Transrectal ultrasonography confirmed 7 (26.9%) recipients to be pregnant 30 days after embryo transfer. Two *rhPA* transgenic goats (BP21 and BP22) were obtained from 7 pregnant recipients derived from donor cells (Fig 3). We obtained 2 F1 transgenic goats through the mating of BP21 with a normal male goat. The results suggested that *rhPA* transgenic cells could support early embryonic development, and the *rhPA* gene could be stably inherited. However, BP22 died because of a difficult labor and edema after cesarean section; BP21 is currently alive and healthy. The protocols for the surgery and embryo transfer were as described earlier [18].

The integration of the *rhPA* gene into the genome of transgenic goats was confirmed by PCR and Southern blot analysis. The *rhPA* transgene was integrated into the genome of the cloned goats BP21 and BP22 (Fig 3). This result was also further verified by Southern blot analysis (Fig 4).

### ELISA for rhPA expression level in transgenic goat milk

**Fig 5:**
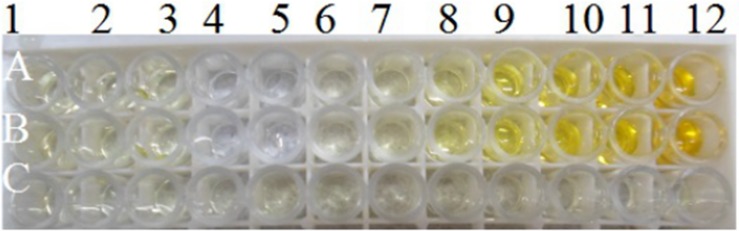
ELISA analysis of partial whey from the milk of the transgenic goat BP21. A1–A3, Whey collected on different days from BP21 (diluted to 1,000 folds); C1–C12: Whey from the F1 transgenic goat; A4, B4: PBS as the negative control; A5, B5: whey from non-transgenic goats as the negative control; A6–B12: reteplase standard (concentrations are 5, 10, 50, 500, 1,000, 2,000, and 4,000 ng/mL, respectively).

**Fig 6:**
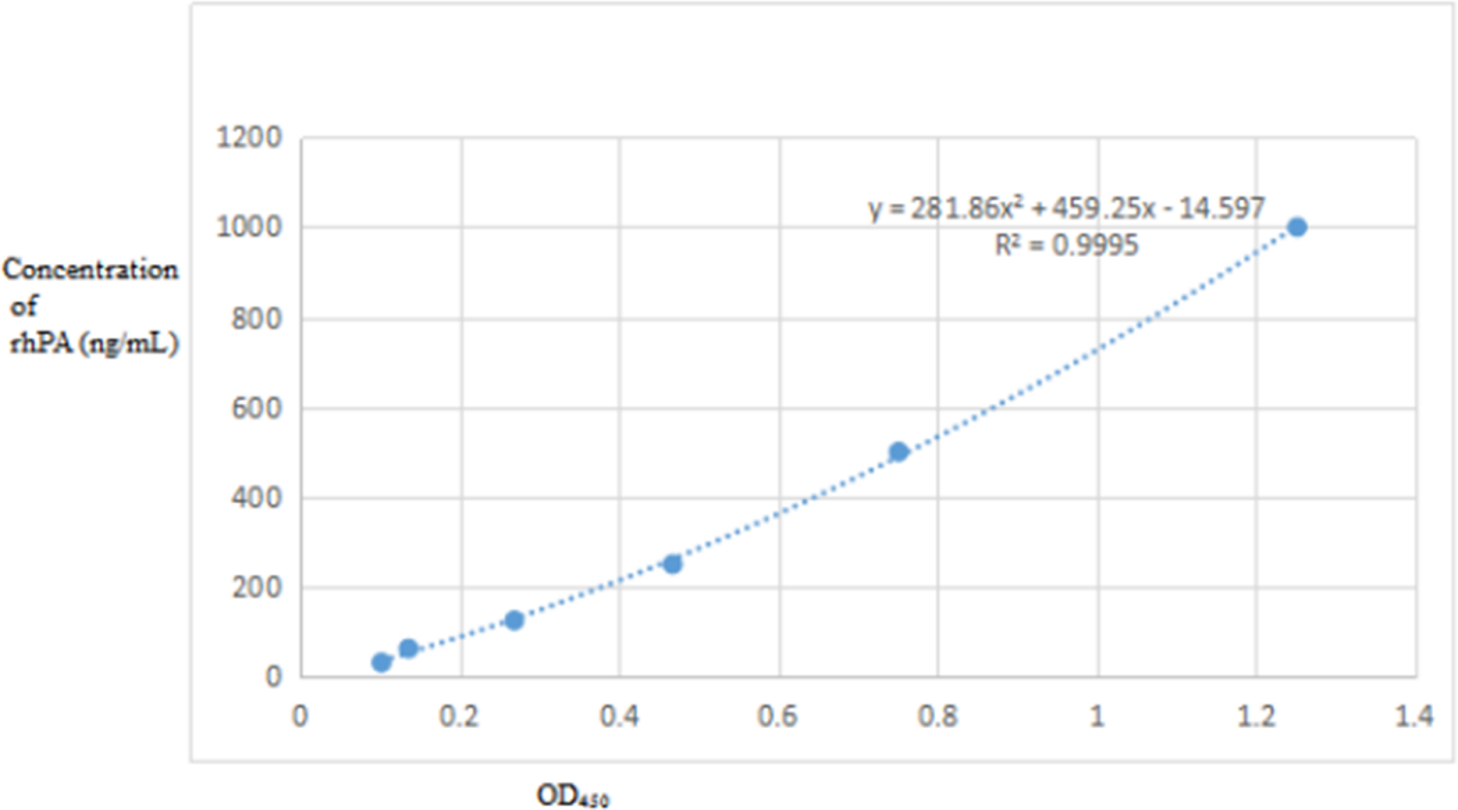
ELISA of milk whey from the transgenic goat BP21 and F1 transgenic goat.

**Fig 7:**
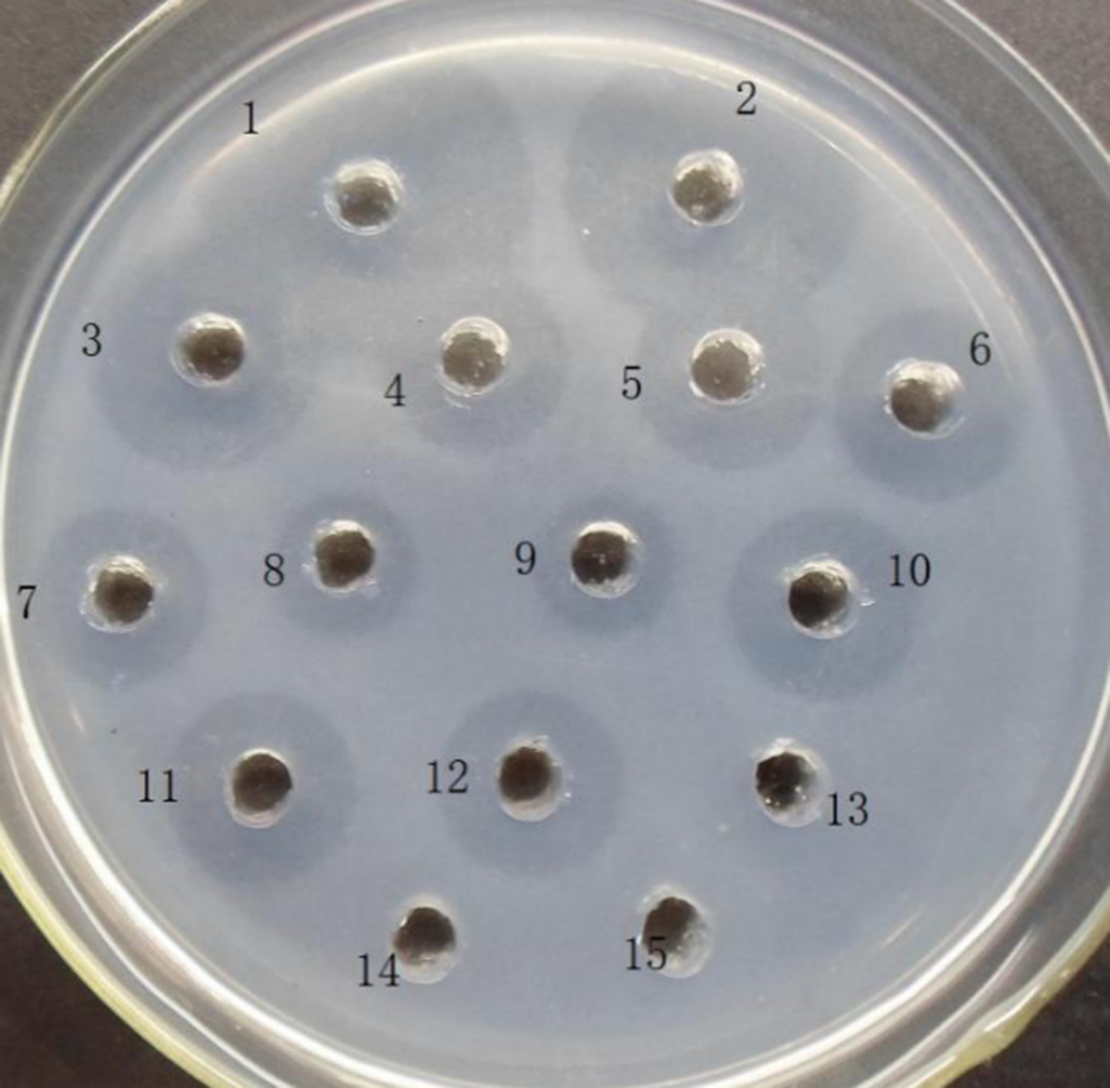
FAPA analysis of partial whey from transgenic goat milk. 1–9: Reteplase standard (concentrations are 2,000, 1,500, 500, 250, 125, 62.5, 31.25, 15.625, and 7.8125 μg/mL, respectively); 10–11: whey collected on different days from the transgenic goat BP21 (diluted 10-folds); 12: whey collected on different days from the F1 transgenic goat (diluted 10-folds); 13, 14: whey from non-transgenic goats as the negative control; 15: PBS as the negative control.

ELISA was used to analyze the rhPA expression level in transgenic goat milk. The rhPA expression level in BP21 and F1 transgenic goat milk was found to be about 78.32 μg/mL (Table 1) and Figs 5 and 6. The results in (Fig 7) and (Table 1) were obtained after the sample was diluted 1,000 times.

**Table 1:** ELISA detection of rhPA in goat milk samples.

Sample serial number	BP21 transgenic goat milk	F1 transgenic goat	6	7	8	9	10	11	12
Concentration of rhPA (ng/mL)	78.3	76.6	5	10	50	500	1000	2000	4000
OD_450_	0.182 ± 0.10	0.179 ± 0.10	0.101 ± 0.05	0.135 ± 0.06	0.155± 0.06	0.268 ± 0.09	0.467 ± 0.03	0.751 ± 0.05	1.252 ± 0.02

### Activity assay of rhPA in the milk of transgenic goats

**Fig 8:**
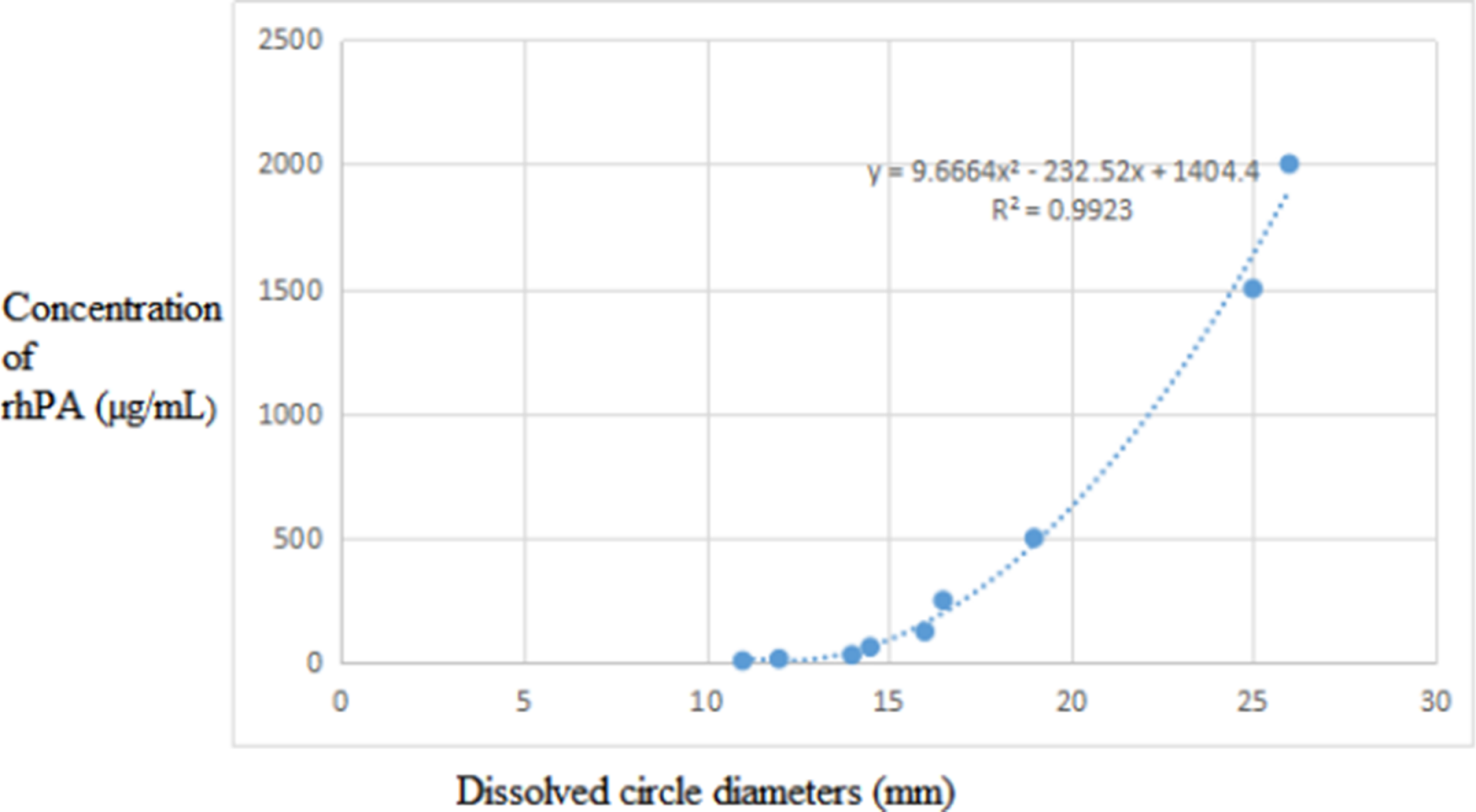
Specific activity of rhPA in the partial transgenic milk samples.

The fibrinolytic activity of rhPA was measured using the size of the thrombolytic transparent circle. The activity analysis (FAPA) showed that the rhPA products expressed by the transgenic goats had fibrinolytic activity *in vitro* (Fig 7) and (Table 2); the activity was equivalent to approximately 1,042 μg/mL of the reteplase standard (Fig 8)[18]. Thrombolytic transparent circles were not observed for non-transgenic goat milk or PBS. This corresponded with the ELISA results (Table 2).

**Table 2:** Specific activity of rhPA in goat milk samples.

Sample serial number	1	2	3	4	5	6	7	8	9	10	11	12
Concentration of rhPA (μg/mL)	2000	1500	500	250	125	62.5	31.3	15.6	7.8	125	125	62.5
Dissolved circle diameters (mm)	26.0	25.0	19.0	16.5	16	14.5	14.0	12.0	11.0	16.0	16.0	14.5

### Analysis of rhPA expression in the milk of transgenic goats using western blot analysis

**Fig 9:**
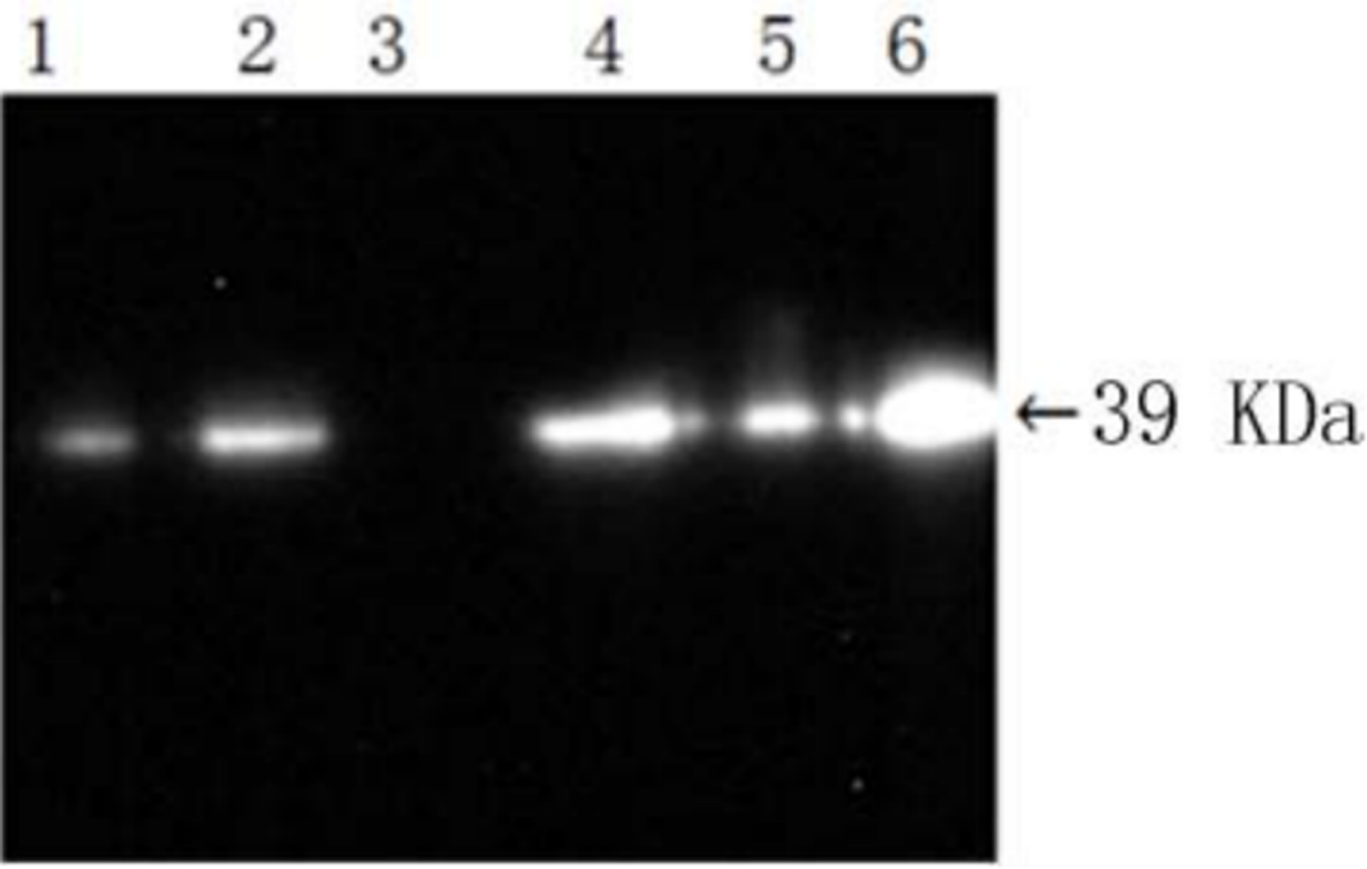
Western blot analysis of partial whey from *rhPA* transgenic goats. 1–2: Whey collected on different days from the BP21 transgenic goat; 3: whey from non-transgenic goats as the negative control; 4, 5: whey collected on different days from the F1 transgenic goat; 6: reteplase as the positive control.

The result is shown as 1 band representing reteplase antigenicity (Fig 9). The molecular weight is about 39 kDa, which corresponds to reteplase. The results demonstrate that *rhPA* transgenic goats can express rhPA.

## Discussion

The aim of this study was to explore the feasibility of producing novel, highly efficient thrombolytic drugs by constructing a mammary gland–specific expression vector BLC14-CMV-rhPA-Neo and developing transgenic goats. To our knowledge, this is the first report on the expression of rhPA by transgenic goats [6, 9], wherein the milk demonstrates high thrombolytic bioactivity. The expression level of rhPA in the milk was about 78.32 μg/mL by ELISA and about 1,042 μg/mL by FAPA, which means the rhPA concentration in the transgenic goat milk reached that of the reteplase reference material, and proved that *rhPA* transgenic goats had good post-translational modification, superior to that of the reteplase reference material [6,9]. This provides an experimental basis and reference guide for establishing a new strain of *rhPA* transgenic goats and large-scale preparation of thrombolytic agents [6].

Studies have shown recombinant protein expression in transgenic animals to have achieved more than 100 times the expression level of prokaryotic expression systems [19–24]. The present FAPA results showed that the rhPA expression in the BP21 goat was about 13.3 times higher than the prokaryotic expression of the reteplase reference. It is likely that goat milk contains some agent that can influence the FAPA or ELISA results, or the rhPA gene integration site in BP21 is not optimum. Hence, the results appear to be a deviation from what was expected [25–27]. This highlights the need for more in-depth studies using purified milk from transgenic animals [28].

In this study, transgenic goats were generated by cloning, whereby the exogenous gene was randomly integrated into the animal genome. It is currently difficult to control the integration locus site and copy number. Different integration locus sites might influence expression level and thrombolytic bioactivity [27, 29–30]. We built a BLC14/rhPA expression vector containing enhancer regulation components, which can enhance rhPA thrombolytic bioactivity. In this study, we only obtained 1 gene integration locus site from transgenic goat milk samples. However, the sample size was small. Therefore, more gene integration sites should be investigated in transgenic goats to evaluate the rhPA expression level and rhPA thrombolytic bioactivity in transgenic goat milk.

According to some reports [14], the hereditary stability of transgenes in transgenic goats is poor. However, in this study, the expression of *rhPA* and the thrombolytic bioactivity were stable in the F1 transgenic goat.

Often, the same recombinant proteins and the same expression vectors differ in expression level in different animals [11, 15]. Such differences might be ascribed to vector construction or locus sites, or the influence of different recombinant proteins on the expression level or stability [11, 15]. Therefore, as the next step in identifying the most ideal expression vector and animal for different recombinant proteins, we should study how to control the transgene locus sites, how to construct an efficient expression vector in transgenic animals, and which recombinant proteins adapt to which expression vector. In future studies, rhPA expression yield and thrombolytic bioactivity must be further improved by optimizing the expression vector construction or other methods [15, 31–38].

## Summary

The mammary gland bioreactor can use a mammary gland–specific expression vector to guide the expression of a foreign gene having specific expression in the mammary glands. Compared to other expression systems such as prokaryotic systems, modification after protein translation can lead to higher biological activity in animal milk. Furthermore, transgenic goat milk is preferable because the structure and function of the rhPA protein is similar to human protein, and it is easy to purify because of the relatively simple ingredients in milk [15, 35–38]. The mammary gland bioreactor has been used for several important proteins, such as lysozyme, human lactoferrin, and human growth hormone, of which some have been used in clinical treatment [39–42].

The *rhPA* transgenic goats obtained in this study showed stable expression of the exogenous gene and demonstrated high rhPA thrombolytic bioactivity in the mammary glands. This research provides a reliable basis for the large-scale production of thrombolytic biological pharmaceuticals in future [6].

In the road ahead, improving the activity and stability of rhPA expression in transgenic goats, as well as enhancing the *in vitro* activity, will be focus areas [6, 14–15].
